# Postnatal depression screening in a paediatric primary care setting in Italy

**DOI:** 10.1186/s12888-017-1205-6

**Published:** 2017-01-25

**Authors:** Antonio Clavenna, Elena Seletti, Massimo Cartabia, Anna Didoni, Filomena Fortinguerra, Teresa Sciascia, Luca Brivio, Daniela Malnis, Maurizio Bonati, on behalf of the Postnatal Depression Study Group

**Affiliations:** 1 Laboratory for Mother and Child Health, Department of Public Health, IRCCS- Istituto di Ricerche Farmacologiche “Mario Negri”, via Giuseppe La Masa 19, Milan, 20156 Italy; 2 Child and Adolescent Neuropsychiatry Unit, Fondazione IRCCS Ca’ Granda - Ospedale Maggiore Policlinico, Milan, Italy; 3 ATS Città Metropolitana di Milano, Milan, Italy; 4 Direzione Socio Sanitaria, ASST Santi Paolo e Carlo, Milan, Italy

**Keywords:** Depression, Postpartum, Screening, Pediatrics, Physicians, Primary Care

## Abstract

**Background:**

Postnatal depression is a non-psychotic depressive disorder that begins within 4 weeks of childbirth and occurs in 13% of mothers and 10% of fathers.

A prospective study with the aim to evaluate the prevalence of postnatal depression by screening parents with the Edinburgh Postnatal Depression Scale (EPDS) in the Italian paediatric primary care setting was performed.

**Methods:**

Mothers and fathers of infants born between 1 February and 31 July 2012, living in Italy’s Milan-1 local health unit area, represented the target population of this pilot study. Parents attending well-child visits at any of the family paediatricians’ offices between 60 to 90 days postpartum were asked to participate in the screening and to fill out the EPDS questionnaire. A cut-off score of 12 was used to identify parents with postnatal depression symptoms.

Maternal and paternal socio-demographic variables and information concerning pregnancy and delivery were also collected.

To investigate the association between screening positivity (dependent variable) and socio-demographic variables and factors related to pregnancy and delivery, a Pearson’s χ2 test was used.

Moreover, a stepwise multivariate logistic regression was carried out to evaluate the risk factors that most influence the probability of suffering from postnatal depression.

**Results:**

In all, 126 out of 2706 (4.7%, 95% CI 3.9–5.5%) mothers and 24 out of 1420 (1.7%, 95% CI 1.0–2.4%) fathers were found to be positive for depressive symptoms. Women with mood disorders and anxiety during pregnancy were at increased risk of postpartum depression (OR 22.9, 95% CI 12.1–43.4).

Only 11 mothers (8.7%) positive to EPDS screening attended a psychiatric service, and for 8 of them the diagnosis of postnatal depression was confirmed.

**Conclusions:**

The prevalence of postnatal depression was lower than previously reported. Routine screening resulted ineffective, since few mothers found positive for depression symptoms decided to attend psychiatric services.

## Background

Postpartum depression (PPD) is a non-psychotic depressive disorder that the Diagnostic and Statistical Manual of Mental Disorders classifies as an Episode of Major Depressive Disorder that begins within 4 weeks of childbirth [1]. The international prevalence of maternal PPD was estimated at 13%, but the data are influenced by several factors such as screening tools, screening period, and country in which the study was conducted [2, 3]. While maternal depression and its influence on child development is well studied [4, 5], there are few studies on paternal depression, whose estimated prevalence is 10.4% [6].

In Italy few studies have been carried out and are characterized by large variability. Their estimates of maternal PPD range from 1.8% to 38.9% [7–15]. Only one study evaluated the prevalence of paternal PDD in Italy, with an estimate of 12.6% in the first month after the birth of the neonate. [10]

Postpartum depression adversely affects mothers, infants, and families [16–20], and the early recognition of PPD symptoms is crucial for the health of the mother and neonate [21].

Several tools were developed for this purpose, and the most widely used is the Edinburgh Postnatal Depression Scale (EPDS) [22]. This is a 10-item, self-reported questionnaire in which women are asked to rate how they have felt in the previous 7 days. Each question is scored 0–3 (resulting range 0–30) [23].

The cut-off score of 12/13 was considered the best threshold for identifying women at risk of PPD, with a sensitivity of 86% and a specificity of 78% [23].

To improve identification of the disease, programmes providing universal screening in the primary care setting have been proposed, but their effectiveness has been debated [24–28].

In Italy, no study has ever evaluated the feasibility of EPDS screening in the paediatric primary care setting. In this regard, a pilot study was performed to evaluate the feasibility of routine screening with EPDS by family paediatricians, to estimate the prevalence of PPD symptoms in mothers and fathers, and to evaluate the influence of maternal and paternal characteristics on the risk of PPD.

## Methods

### Study design and procedures

An observational prospective study was performed in the area covered by the Milan-1 local health unit (ASL Milano 1, now part of the ATS della Città Metropolitana di Milano), in northern Italy’s Lombardy Region.

Postnatal depression screening was proposed to the parents of infants born between 1 February and 31 July 2012 who attended the second well-child visit performed by the family paediatrician (FP).

Italian FPs routinely perform periodic well-child visits to monitor the growth and wellbeing of children. The first visit is usually scheduled around 30–45 days after birth, and the second between 60 and 90 days afterwards. The timing of the second well-child visit coincides with the period in which the risk of postpartum depression is greatest [2], it is consistent with the period chosen for questionnaire administration by Benvenuti et al in the validation study of the Italian version of EPDS [8], and therefore represents an adequate time frame for screening, without implying an additional burden for FPs and parents.

During the first well-child visit FPs explained the aim of the study to parents, provided them with an information leaflet, and asked them to attend the following visit.

The EPDS questionnaire was given to mothers or fathers, or both, during the 2nd well-child visit. Parents were asked to self-complete the questionnaire in the FP’s waiting room and to return it to the FP. Privacy was guaranteed to parents when filling in the questionnaire and parents were asked to complete the questionnaire separately. The Italian version of EPDS, for which the cross-cultural equivalence with the original one was established [8], was used in the study. It was first validated by Carpiniello et al in 1997 [9], and, subsequently, by Benvenuti et al, who estimated the maximum positive predictive value (PPV) using 12/13 points as a threshold (PPV = 90.9%), with a specificity of 98.9% and a sensitivity of 55.6% [8].

Socio-demographic variables of the mothers and fathers (age, educational level, employment, marital status) were collected. Mothers were also asked if they perceived delivery as difficult or problematic, if they had mood disorders and/or excessive anxiety during pregnancy, if they attended antenatal courses, and if they received information on PPD during the course.

Data on the child (date of birth, gestational age at birth, birth weight, and type of pregnancy and delivery) were collected by the FP on his/her case report form.

Mothers and fathers were considered positive for postpartum depression if the EPDS score was 13 or more. These parents were referred to one of three of the LHU’s referral psychiatric services for postpartum depression care, using a protocol defined before the start of the study.

Mothers and fathers who were negative at the time of EPDS screening, and who subsequently developed depressive symptoms, had the possibility to attend psychiatric services through referral by general practitioners or through self-referral.

Psychiatrists and psychologists of the psychiatric services were asked to inform the FP and the coordinating centre of whether the diagnosis of PPD was confirmed or not.

Before the start of the study, FPs received specific training on the symptoms and effects of postpartum depression, on the use of the EPDS, and on the referral protocol.

The study was approved by the Milan LHU’s Ethics Review Board. Data were analysed using an anonymous patient code.

Written consent was obtained by all parents participating in the study.

### Data analysis

Prevalence was estimated as the number of positive mothers and fathers over the number of mothers/fathers screened.

To investigate the association between screening positivity (dependent variable) and socio-demographic variables and factors related to pregnancy and delivery, a Pearson’s χ^2^ test was used and odds ratios (OR) with 95% CIs were calculated.

Moreover, a stepwise multivariate logistic regression was carried out in order to evaluate the risk factors that most influence the probability of having PPD symptoms. All variables with at least 10% significance (*p* < 0.10) with the univariate analysis were included in the model.

## Results

During the 6 month observation period, 4206 infants were born in the Milan-1 LHU area, 3705 of whom were cared for by one of the 117 FPs involved in the study. Mothers and/or fathers of 2727 (74%) newborns agreed to participate in the study. The mean number of infants per FP with at least one parent who underwent screening with the EPDS was 23 (range 4–53).

Parent characteristics are reported in Table 1. The mean age was 33.5 years (SD 4.8) for the mothers and 36.3 years (SD 5.8) for the fathers. In all, 68% of mothers and 62% of fathers were 30–39 years old, and 7% were single-parents.

**Table 1:** Characteristics of mothers and fathers participating in the study

Variable	Mothers N (%)	Fathers N (%)
Age (years)		
≤ 24	144 (5.7)	26 (1.9)
25–29	468 (18.3)	155 (11.3)
30–34	896 (35.1)	386 (28.2)
35–39	839 (33.0)	465 (34.0)
≥ 40	202 (7.9)	336 (24.6)
Education		
Primary school	466 (17.4)	424 (30.1)
Secondary School	1354 (50.5)	681 (48.4)
Degree/PhD	861 (32.1)	303 (21.5)
Occupation		
Yes	2247 (84.1)	1387 (98.2)
No	424 (15.9)	25 (1.8)
Marital status		
Married	2492 (92.5)	1319 (93.1)
Single	202 (7.5)	98 (6.9)
Primiparous		
Yes	1407 (52.7)	569 (40.7)
No	1262 (47.3)	828 (59.3)
Pregnancy Course		
Normal	2441 (91)	1278 (90.5)
Pathological	241 (9)	134 (9.5)
Delivery Course		
Regular	2056 (77)	715 (74.8)
Difficult	433 (16.2)	161 (16.9)
Problematic	183 (6.8)	79 (8.3)
Type of delivery		
Vaginal	1834 (68.9)	960 (69.1)
Caesarean	827 (31.1)	429 (30.9)
Weight		
≤ 2500 g	168 (6.2)	87 (6.1)
> 2500 g	2532 (93.8)	1333 (93.9)
Weeks of gestation		
< 37	180 (6.7)	89 (6.3)
≥ 37	2513 (93.3)	1327 (93.7)
Antenatal course		
Yes	1717 (64.6)	n.a.
No	942 (35.4)	
Information about PPD		
Yes	1272 (74.0)	n.a.
No	445 (26.0)	
Mood changes		
Yes	1199 (45.0)	n.a.
No	1465 (55.0)	
Excessive Anxiety		
Yes	343 (13.5)	n.a.
No	2190 (86.5)	

n.a.: not applicable

A total of 126 out of 2706 (4.7%, 95% CI 3.9–5.5%) mothers and 24 out of 1420 (1.7%, 95% CI 1.0–2.4%) fathers resulted positive to the EPDS test.

In 8 out of 1410 parent couples (0.6%) both mother and father were positive to the questionnaire.

Results of univariate analyses evaluating the effect of maternal characteristics, and of variables related to the neonate, pregnancy, and delivery, on the risk of having PPD symptoms are shown in Table 2. An increased risk of maternal depressive symptoms was strongly associated with a history of mood (OR 7.2, 95% CI 4.5–12.15, *p* < 0.001) and/or anxiety disorder (OR 9.6, 95% CI 6.5–14.3, *p* < 0.001) during pregnancy.

**Table 2:** Characteristics of mothers with PPD symptoms

	N (%)	crude O.R.	95% CI	*p*-value
Age (years)				
≤ 24	14 (9.7)	1		
≥ 25	105 (4.4)	0.47	0.26 to 0.91	0.006
Education				
Primary School	29 (6.2)	1		
Secondary School/Degree	94 (4.2)	0.67	0.43 to1.03	0.06
Employment				
Yes	83 (3.6)	1		
No	37 (8.7)	2.49	1.65 to 3.71	<0.0001
Marital status				
Married	109 (4.3)	1		
Single	17 (8.4)	2.00	1.18 to 3.42	0.009
Primiparous				
Yes	71 (5.0)	1		
No	53 (4.2)	0.82	0.57 to 1.19	0.30
Pregnancy Course				
Normal	107 (4.4)	1		
Pathological	18 (7.4)	1.76	1.02 to 2.91	0.03
Delivery Course				
Regular	69 (3.3)	1		
Difficult/Problematic	55 (8.9)	2.82	1.96 to 4.07	<0.0001
Type of delivery				
Vaginal	74 (4.0)	1		
Caesarean	51 (6.1)	1.56	1.08 to 2.91	0.02
Weight				
≤ 2500 g	10 (5.9)	1		
> 2500 g	115 (4.5)	1.33	0.65 to 2.51	0.40
Weeks of gestation				
< 37	2 (1.1)	1		
≥ 37	113 (4.5)	4.19	1.03 to 17.10	0.03
Antenatal course				
Yes	81 (4.7)	1		
No	44 (4.7)	0.99	0.68 to 1.44	0.97
Information about PPD				
Yes	49 (3.8)	1		
No	32 (7.2)	1.93	1.22 to 3.06	0.0615
Mood changes				
No	19 (1.3)	1		
Yes	104 (8.6)	7.22	4.48 to 12.15	<0.0001
Excessive Anxiety				
No	50 (2.3)	1		
Yes	63 (18.3)	9.6	6.50 to 14.28	<0.0001

Other variables found to be associated with the possibility of presenting symptoms were young age, being a single parent, unemployment, and factors related to the pregnancy such as type of delivery, the course of pregnancy, and the maternal perception of the delivery.

In the logistic regression model the combined presence of mood disorders and anxiety during pregnancy was associated with an OR of 22.9 (95% CI 12.1–43.4) of developing PPD symptoms, while having only one of them decreased the OR to 4.7 (95% CI 2.5–8.9).

The risks for unemployed women and for single parents were, respectively, 2.4 (95% CI 1.5–3.8) and 1.8 (95% CI 1.0–3.4) times greater than the respective reference categories. Having perceived the delivery as difficult or problematic was associated with an OR of 2.3 (95% CI 1.2–4.5).

The univariate analyses found that the variables associated with an increased risk of PPD symptoms in fathers were being a single parent (OR 4.5; 95% CI 1.7–11.6), having a low education level (OR 3.4; 95% CI 1.5–7.6), not becoming father for the first time (OR 2.9; 95% CI 1.3–7.4), and having a partner with both mood and anxiety disorders during pregnancy (OR 5.9; 95% CI 2.1–16.6) (Table 3).

**Table 3:** Characteristic of fathers with PPD symptoms

	N (%)	crude O.R.	95% CI	*p*-value
Age (years)				
≤ 29	6 (3.3)	2.2	0.8–5.6	0.12
≥ 30	18 (1.5)	1		
Education				
Primary School	13 (3.1)	3.4	1.5–7.6	0.013
Secondary School/Degree	11 (1.1)	1		
Employment				
Yes	23 (1.7)	1		
No	1 (5.9)	3.9	0.2–23.6	0.36
Marital status				
Married	18 (1.4)	1		
Single	6 (6.1)	4.5	1.3–7.4	0.005
First child				
Yes	8 (1.0)	1		
No	16 (2.8)	2.9	1.3–7.4	0.011
Pregnancy Course*				
Normal	21 (1.6)	1		
Pathological	3 (2.2)	1.4	0.3–4.3	0.49
Delivery Course*				
Regular	14 (1.3)	1		
Difficult/Problematic	10 (2.9)	2.2	0.9–5.0	0.10
Type of delivery*				
Vaginal	16 (1.9)	1		
Caesarean	8 (1.7)	0.9	0.4–2.2	0.77
Birth weight (newborn)				
≤ 2500 g	2 (2.3)	1.4	0.2–5.2	0.65
> 2500 g	22 (1.7)	1		
Weeks of gestation				
< 37	1 (1.1)	0.6	0.03–3.5	1.00
≥ 37	23 (1.7)	1		
Antenatal course*				
Yes	13 (1.4)	1		
No	11 (2.3)	1.7	0.6–3.7	0.35
Information about PPD*				
Yes	10 (1.5)	1		
No	3 (1.3)	0.9	0.2–3.0	1.00
Mood changes*				
No	15 (1.2)	1		
Yes	9 (2.3)	1.9	0.8–5.0	0.17
Excessive Anxiety*				
No	16 (1.4)	1		
Yes	8 (4.4)	3.2	1.3–7.7	0.02

*information provided by the partner (mother)

The adjusted OR for paternal PPD symptoms associated with having a partner who reported “changes in mood and excessive anxiety” during pregnancy was 4.4 (95% CI 1.6–12.5). Moreover, being a single parent increased the risk 4.3 fold (95% CI 1.6–11.9). Becoming fathers not for the first time was associated with an adjusted OR for PPD of 2.7 (95% CI 1.1–6.6).

Of the 126 mothers who resulted positive to the EPDS screening, only 11 (8.7%) attended the LHU’s psychiatric services: for 8 of them the diagnosis of postpartum depression was confirmed.

Eight mothers who resulted negative to the EPDS screening attended the psychiatric services and were all diagnosed with postpartum depression.

In all, for 8 out of the 19 (42%) mothers seen by a mental health professional, the results of the EPDS screening and the clinical evaluation matched.

None of the 24 fathers positive to EPDS screening attended the LHU’s psychiatric services.

## Discussion

This study had the largest number of participating parents in Italy, and was the first to evaluate the routine screening of mothers and fathers with the EPDS in the Italian paediatric primary care setting.

The prevalence observed in our sample (4.7% of mothers) was generally lower than that reported at the national and international levels [2, 7–15]. In Italy, the prevalence of PPD at 3 months, estimated with the EPDS with a cut-off ≥13, ranged between 2.7% and 38.9% [7, 8, 11, 14, 15].

These differences may be related to the characteristics of the women (e.g. socio-demographic, economic, and cultural factors), the setting (geographical or urban versus rural), or the study design (e.g. sample size, inclusion criteria, timing of the screening, and observation period).

A study that screened a cohort of women between the 6th and 12th week after birth in the Bergamo Province (about 60 km away from the Milan-1 LHU area) found a slightly greater prevalence (7.1%) than that found in the Milan-1 LHU area [15]. The screening performed in the Bergamo Province enrolled only women who attended antenatal courses in three hospitals, and included also migrant mothers. These factors may partly explain the different estimates between two closely located settings, with similar socioeconomic and demographic characteristics.

The prevalence of depressive symptoms in fathers was very low. Only one Italian study evaluated the prevalence of PPD in fathers, with an estimate of 12.6% [10]. It should, however, be underlined that the screening was performed between 15 and 20 days after the birth of the newborn, and an EPDS cut-off score of 7 was used. Several factors could explain the low prevalence of symptoms in fathers, for example the greater reluctance in reporting distress, gender differences in manifesting depressive symptoms, and, consequently, the potential need for different screening tools or cut-off scores for mothers and fathers, and differences in the onset of depressive symptoms, which may be delayed in fathers [29].

The presence of mood disorders and/or anxiety during pregnancy resulted as the main determinant of the risk of depressive symptoms in mothers. Other factors that increased the risk of PPD symptoms were unemployment, the lack of partner support, and the perception of delivery as a problematic event. These findings are consistent with previous studies [10, 15, 30, 31].

A partner who had mood disorder and/or anxiety during pregnancy is a major risk factor also for paternal PPD. Several studies reported that having a partner with elevated depressive symptoms or depression was the most common correlate of paternal depression [32], and a positive, and moderate in size (*r* = 0.308; 95% CI, 0.228–0.384), correlation between paternal and maternal depression was found in the meta-analysis by Paulson and Dazemore [6].

Taking into account that family paediatricians in Italy are the guardians of the health of children and their families, well-child visits (in particular the second one) could represent a good occasion to perform universal screening with the EPDS. The percentage of mothers (73%) and fathers (38%) who participated in the screening supports this possibility. This percentage is estimated using the total number of infants in the cohort (3705) as the denominator. In this regard, a lower rate of paternal participation in the screening is expected, since only 10% of fathers usually attend the second well-child visit (data provided by the FPs before the start of the study).

The effectiveness of universal screening in the primary care setting is widely debated [26–28, 33].

The usefulness of screening has been advocated in the United States [26, 33, 34], while a universal screening programme was found not to be effective in one Australian rural shire [24], and routine screening was reported as not cost-effective in the UK, mainly due to the potential additional costs of managing women incorrectly diagnosed as depressed [27].

In particular, the lack of sound evidence regarding the cost-effectiveness of EPDS screening is a relevant barrier to its adoption in universal screening programmes in primary care, and more research is needed in this area [35]. Unfortunately, we cannot provide any information on the costs of the screening, but the costs related to the management of false positives do not seem to represent a major problem in the Italian setting.

In our study, few mothers and no fathers agreed to attend a psychiatric service dedicated to the management of postpartum depression. Our findings indicate that the most relevant problem of a PPD screening performed in the Italian paediatric primary care setting could be the scant access to specialist evaluation.

From this point of view, routine screening of parents, alone, does not seem useful: it should be part of a wider initiative involving the target population, psychiatric services, and primary care family physicians and paediatricians.

### Strengths and limitations

The main strength of our pilot study was the possibility to screen a significant number of parents of a cohort of newborns in a homogeneous setting, covering 73% of mothers.

There were, however, some limitations. Only parents with a good comprehension of the Italian language were included, and results may not be applicable to migrant parents. Moreover, the primary aim of the study was not to evaluate the predictors of PPD, so only a few socio-demographic variables were collected, and data concerning self esteem, childcare stress, life stress, parental expectations, parental relationships, and infant temperament were not recorded or analysed.

Unfortunately, we were not able to collect data on why parents who resulted positive to the screening did not attend psychiatric services or how mothers who were negative to the screening attended them (e.g. referral by general practitioner versus self-referral).

Finally, it is possible that a few parents positive to EPDS screening attended a private psychiatric service and could therefore not be monitored for confirmation of the diagnosis, resulting in an underestimation of the percentage of mothers and fathers who seek help from a mental health professional.

## Conclusions

In conclusion, this pilot study indicates that a routine screening with EPDS by family paediatricians is feasible and can cover a significant proportion of mothers and fathers. This screening, however, does not seem to be effective in identifying parents with postpartum depression, if not as a part of a wider initiative involving the target population and psychiatric services.

## Abbreviations

EPDS: Edinburgh postnatal depression scale
FP: Family paediatrician
LHU: Local health unit
NHS: National health service
PPD: Postpartum depression
